# A Novel Strain of Tomato Leaf Curl New Delhi Virus Has Spread to the Mediterranean Basin

**DOI:** 10.3390/v8110307

**Published:** 2016-11-11

**Authors:** Isabel M. Fortes, Sonia Sánchez-Campos, Elvira Fiallo-Olivé, Juan A. Díaz-Pendón, Jesús Navas-Castillo, Enrique Moriones

**Affiliations:** Institute for Mediterranean and Subtropical Horticulture “La Mayora” (IHSM-UMA-CSIC), Consejo Superior de Investigaciones Científicas, La Mayora Experimental Station, 29750 Algarrobo-Costa, Málaga, Spain; ifortes@eelm.csic.es (I.M.F.); soniasc@eelm.csic.es (S.S.-C.); efiallo@eelm.csic.es (E.F.-O.); diazpendon@eelm.csic.es (J.A.D.-P.); jnavas@eelm.csic.es (J.N.-C.)

**Keywords:** *Begomovirus*, cucurbits, genetic diversity, recombination, tomato, Tomato leaf curl New Delhi virus

## Abstract

Tomato leaf curl New Delhi virus (ToLCNDV) is a whitefly-transmitted bipartite begomovirus (genus *Begomovirus*, family *Geminiviridae*) that causes damage to multiple cultivated plant species mainly belonging to the *Solanaceae* and *Cucurbitaceae* families. ToLCNDV was limited to Asian countries until 2012, when it was first reported in Spain, causing severe epidemics in cucurbit crops. Here, we show that a genetically-uniform ToLCNDV population is present in Spain, compatible with a recent introduction. Analyses of ToLCNDV isolates reported from other parts of the world indicated that this virus has a highly heterogeneous population genetically with no evident geographical, plant host or year-based phylogenetic groups observed. Isolates emerging in Spain belong to a strain that seems to have evolved by recombination. Isolates of this strain seem adapted to infecting cucurbits, but poorly infect tomatoes.

## 1. Introduction

Begomoviruses are emergent pathogens widely distributed in tropical and temperate regions worldwide and are a serious threat to diverse economically-important crops [1,2]. The genus *Begomovirus* is the largest of the seven genera currently included in the family *Geminiviridae* (*Becurtovirus*, *Begomovirus*, *Curtovirus*, *Eragrovirus*, *Mastrevirus*, *Topocuvirus* and *Turncurtovirus*) classified based on its host range, insect vector and genomic characteristics [3,4]. Begomoviruses can have bipartite or monopartite genomes. The genome of bipartite begomoviruses consists of two circular single-stranded DNA molecules of about 2.5–2.7 kb, referred to as DNA-A and DNA-B, which are encapsidated within twin-shaped (“geminate”) virions. Monopartite begomoviruses have a single-genomic DNA that is homologous to the DNA-A of the bipartite members of the family. Begomoviruses are transmitted in nature by the whitefly *Bemisia tabaci* Genn. (*Hemiptera*: *Aleyrodidae*) and infect dicotyledonous plant species [3].

Tomato leaf curl New Delhi virus (ToLCNDV) is an economically-important bipartite begomovirus reported to cause damage in cultivated plant species of the *Solanaceae*, including tomato (*Solanum lycopersicum* L.), potato (*Solanum tuberosum* L.), chili pepper (*Capsicum annuum* L.) and eggplant (*Solanum melongena* L.) [5,6,7,8,9], and *Cucurbitaceae*, including melon (*Cucumis melo* L.), pumpkin (*Cucurbita maxima* L.), luffa (*Luffa acutangula* (L.) Roxb.) and cucumber (*Cucumis sativus* L.) [10,11,12,13,14]. In tomato, the virus causes severe stunting of plants, which exhibit upward and downward rolling and crinkling of the leaves, interveinal yellowing and vein clearing, with infected plants presenting partial or complete yield loss depending on the growth stage at which infection occurs [15]. ToLCNDV is widespread and more prevalent in northern India, where it is an important constraint to tomato production [15,16]. Reports of ToLCNDV presence were limited to the Indian subcontinent and other countries in Asia until 2012, when it was first detected in the western Mediterranean Basin infecting zucchini squash (*Cucurbita pepo* L.), melon and cucumber crops in southern Spain [17]. Subsequently, severe epidemic outbreaks of the disease were associated with ToLCNDV and caused serious economic losses to greenhouse and open-field cucurbit crops [18]. Recently, this virus has been detected infecting tomato crops in the same area [19]. Moreover, further spread has occurred with recent reports of ToLCNDV in Tunisia infecting cucurbits (melon, cucumber and zucchini squash) and in Italy infecting zucchini squash, with isolates closely related to those detected in Spain [20,21].

The presence of ToLCNDV in the Mediterranean Basin represents a new threat for economically-important cucurbit crops, as well as for tomato production. Knowledge about the genetic diversity and host range of isolates associated with the epidemics is essential for durable control. Genetic resistance to ToLCNDV in cucurbits has been identified and is being introgressed into commercial varieties [22]. In tomato, genetic resistance to begomoviruses has been deployed in the region to control tomato yellow leaf curl disease (TYLCD)-associated viruses [23], and effectiveness against the recently-emerged ToLCNDV isolates needs to be studied. In this report, we studied the genetic variability of ToLCNDV isolates in Spain and their phylogenetic relationship with isolates reported from other parts of the world. Furthermore, an infectious clone was obtained for a Spanish isolate of ToLCNDV that helped to study its host range and biology in tomato. The results indicated that a genetically-homogeneous ToLCNDV population seems to be present in Spain with isolates poorly adapted for infecting tomato, but efficient at infecting plants of cucurbit species. Genetic analyses indicated that isolates of a new strain of ToLCNDV that putatively diverged by recombination are spreading in Spain. The ToLCNDV species is genetically complex, but no phylogenetic groups clearly associated with geographical, temporal or host plant species were detected.

## 2. Materials and Methods

### 2.1. Field Sampling

As summarized in Table 1, surveys were carried out during three seasons in 2013–2015 in commercial crops of the provinces of Málaga, Almería and Murcia (southern and southeastern Spain). A total of 83 young leaf samples were collected from symptomatic zucchini squash and melon plants (one sample per plant) at the harvest growth stage. Plants exhibited leaf curl disease-like symptoms, composed of severe mosaic, vein swelling and curling in young leaves, plant stunting and fruit skin roughness similar to those caused by ToLCNDV [17]. Furthermore, 112 samples were collected from plants of commercial tomato crops present near the ToLCNDV-infected zucchini squash or melon crops, which presented begomovirus-like infection symptoms, such as stunting, yellowing and/or curling in apical leaves. With the exception of zucchini squash crops of Murcia, all crops sampled were in fields under protected conditions (plastic houses or net houses). The presence of *B. tabaci* infesting plants was observed in all cases.

### 2.2. Sample Analysis and Genetic Diversity Study

Field samples were analyzed for the presence of ToLCNDV by hybridization of squash blots from petiole cross-sections made on positively-charged nylon membranes (Roche Diagnostics, Mannheim, Germany). Based on ToLCNDV clones previously obtained from a zucchini squash plant [17], a digoxigenin (DIG)-labelled DNA probe was prepared by polymerase chain reaction (PCR) as described by Navas-Castillo et al. [24] using the primer pair MA1788 (5′-CGTGTCGTTTCGATCTGGTGTC-3′) and MA1789 (5′-GTTTGTGGATCTAAACTTGGTGAG-3′) designed for the intergenic region (IR) of the DNA-A component of the ToLCNDV isolate (Spain-MU-8.1-Squash-2012) (ToLCNDV-[ES-MU-8.1-Sq-12]) (GenBank Accession Number KF749224) [17]. Hybridization analysis of squash blots was performed at high stringency conditions (hybridization at 65 °C followed by washing steps with 0.1× Saline sodium citrate [SSC], 0.1% Sodium dodecyl sulfate [SDS] at 65 °C). Furthermore, squash blot hybridization to check for the presence of other begomoviruses was performed for tomato samples using a mixture of DIG-labelled probes specific to the begomoviruses associated with TYLCD also present in the regions surveyed, as described by García-Andrés et al. [25].

Sequence variability among the ToLCNDV isolates present in samples that exhibited a positive reaction in hybridization analyses was analyzed by rolling circle amplification (RCA) with φ29 DNA polymerase combined with the restriction fragment length polymorphism (RFLP) using the restriction enzymes *Hpa*II and *Alu*I that have recognition sequences specified by four nucleotides. For this, total DNA from ToLCNDV-positive samples was extracted using a Cetyltrimethylammonium bromide (CTAB)-based purification method [26], and viral DNA was amplified by RCA (TempliPhi kit, GE Healthcare, Little Chalfont, UK) [27]. RCA products were then digested with *Hpa*II or *Alu*I, and restriction products were analyzed in a 1.5% agarose electrophoresis gel.

### 2.3. Phylogenetic Analysis

The analysis of phylogenetic relationships among isolates of ToLCNDV was performed based on the full-length DNA-A sequences available in databases. A total of 126 full-length ToLCNDV DNA-A sequences were downloaded from databases (all of the isolates described as ToLCNDV, available as of 3 May 2016, in the NCBI-GenBank database and/or listed by the *Geminiviridae* Study Group of the International Committee on Taxonomy of Viruses (ICTV; Supplementary Materials Table S1). Sequences were aligned by using the Multiple Sequence Comparison by Log-Expectation (MUSCLE) computer software [28], and the phylogenetic relationships were represented by means of a tree built using the neighbor-joining method based on the Tamura–Nei model available in the Molecular Evolutionary Genetics Analysis (MEGA) 5.0 program [29]. Pairwise nucleotide identity comparisons were calculated using the Sequence Demarcation Tool program (SDT; Version 1.2) [30] with the MUSCLE [28] option for sequence alignment.

### 2.4. Recombination Analysis

Detection of possible recombinant sequences, localization of putative recombination breakpoints and identification of likely parents involved in the genetic exchange was carried out by using the Recombination Detection Program 4 (RDP4) [31] comprised by RDP, GENECONV, BOOTSCAN, MAXIMUM CHI SQUARE, CHIMAERA, SISCAN and 3SEQ recombination detection methods. The analysis was performed with default settings for the different detection methods and a Bonferroni-corrected *p*-value cut-off of 0.05. Only recombination events detected with three or more methods were considered. The selection of sequences for RDP4 was made based on the similarity analysis performed with the SimPlot 3.5.1 program with the Kimura 2-parameter distance model to compare the DNA-A sequence of ToLCNDV isolates that differ with reported ones at the strain threshold level with that of the isolate recognized by the ICTV as the type isolate for ToLCNDV species, ToLCNDV-[BG-Jes-Svr-05] (GenBank AJ875157, Supplementary Materials Table S1) [3]. Portions of sequences that diverged from that of the type isolate were then used for sequence similarity search using the Basic Local Alignment Search Tool (BLAST) [32] to localize putative parent relatives to be included in the RDP4 analysis.

### 2.5. Construction of Infectious Clones and Agroinoculation

ToLCNDV infectious clones were prepared from DNA-A and DNA-B genomic components of the isolate ToLCNDV-[ES-Alm-661-Sq-13] previously cloned and sequenced by our group [17]. Full-length DNAs (~2.7 kbp) were excised from the cloning vector pBluescript II SK (+) (Stratagene, La Jolla, CA, USA) by digestion with the restriction enzyme *Bam*HI. Viral DNA was eluted from a 0.8% agarose electrophoresis gel using the Montage Gel Extraction Kit according to the manufacturer’s instructions (Millipore Corporation, Bedford, MA, USA), recircularized by a standard ligation reaction with T4 DNA ligase (Roche Diagnostics, Mannheim, Germany) and subjected to RCA amplification. Amplification products obtained were partially digested with *Bam*HI, and tandem repeat fragments were agarose-gel purified as described above and subcloned into the *Bam*HI cloning site of pCAMBIA 0380 vector (Cambia, Canberra, Australia). Head-to-tail dimeric constructs obtained for DNA-A and DNA-B were named ND-AL-661-2.0-A and ND-AL-661-2.0-B, respectively, and were transferred to *Agrobacterium tumefaciens* strain C58C1. For pepper inoculation experiments, these constructs were also transferred to *A. tumefaciens* strains LBA4404 and EHA105. For *A. tumefaciens*-mediated inoculation (agroinoculation), liquid cultures of *A. tumefaciens* containing ND-AL-661-2.0-A and ND-AL-661-2.0-B were pelleted and resuspended in agroinoculation buffer (10 mM MgCl_2_, 10 mM 2-(*N*-Morpholino)ethanesulfonic acid (MES) pH 5.8 and 2.25 mM acetosyringone) to a final OD_600_ of 1.0, and equal volumes of DNA-A and DNA-B cultures were mixed before stem puncture inoculation. Inoculated plants were maintained in an insect-proof growth chamber (26 °C day and 18 °C night, 70% relative humidity, with a 16-h photoperiod at 250 μmol·s^−1^m^−2^ photosynthetically-active radiation) until the analysis and observation of symptom development.

The presence of the DNA-A and DNA-B components in agroinoculated plants was confirmed by squash blot hybridization. DIG-labelled DNA probes specific to each viral component were prepared as described above by using the clones previously obtained [17] and the primers pairs MA1933 (5′-GGATCCATTATTGCACGAATTTCCG-3′) and MA1934 (5′-CATAGGGGCTGTCGAAGTTGAGCC-3′), based on the sequence of the DNA-A component of the isolate ToLCNDV-[ES-Alm-661-Sq-13] (GenBank KF749223), and MA1935 (5′-GCTTTTCCTTCTCCTTATTCCACTC-3′) and MA1936 (5′-CCGTAAAGTCCATTTGTTTGAACAAC-3′), based on the sequence of the DNA-B component of the same isolate (GenBank KF749226).

### 2.6. Host Range Study

A host range study was performed by *A. tumefaciens*-mediated inoculation of the dimeric infectious clones ND-661-2.0-A and ND-661-2.0-B of ToLCNDV-[ES-Alm-661-Sq-13] as described above. In the assays, plants included were of *Cucurbitaceae* (zucchini squash cultivars Milenio (Fitó S.A., Spain) and Afrodita (Syngenta, Spain); Melon Accession Number C-278 (La Mayora-CSIC germplasm bank); Watermelon Accession Number SV80287 (La Mayora-CSIC germplasm bank); and cucumber cultivars Pacer (Sluis and Groot, Spain) and Bell Puig (Fitó S.A.)); *Solanaceae* (tomato cultivars Moneymaker, Fortuna, Gardener’s Delight, Rondeño and Marmande (La Mayora-CSIC germplasm bank); quasi-isogenic homozygous TYLCD susceptible ty-1S (*ty-1*/*ty-1*); and heterozygous TYLCD-resistant Ty-1 F_1_ (*Ty-1*/*ty-1*) tomato lines (kindly provided by María José Díez, Universidad Politécnica de Valencia, Spain); jalapeño and California Wonder pepper types (La Mayora-CSIC germplasm bank)) and *Fabaceae* (common bean cultivar Donna (Nunhems, Spain)) cultivated species; as well as of a wild species (*Solanum nigrum* L.) associated with these crops in the surveyed area. Plants of the experimental hosts *Nicotiana benthamiana* Domin, *N. tabacum* L. cultivar Xanthi and *N. glutinosa* L. of the family *Solanaceae* also were tested, with plants of *N. benthamiana* included as the susceptible control. In the case of pepper, a recalcitrant species to agroinfection, clones ND-661-2.0-A and ND-661-2.0-B transferred to *A. tumefaciens* strains LBA4404 and EHA105 [33] in addition to strain C58C1 were also tested. Control inoculations in tomatoes, zucchini squash cultivar Milenio and *N. benthamiana* were performed using infectious clones of DNA-A (GenBank U15015) and DNA-B (GenBank U15017) components of an Indian isolate of ToLCNDV, (India-New Delhi-Severe-1992) (ToLCNDV-[IN-ND-Svr-92]), collected in New Delhi from tomato [5] (kindly provided by Supriya Chakraborty, School of Life Sciences, Jawaharlal Nehru University, New Delhi, India). Inoculations were performed under confined conditions in an insect-proof growth chamber (see growth conditions above) appropriate for exotic virus containment. In these inoculation studies, we included the commercial tomato hybrid cultivar Anastasia (Seminis Vegetable Seeds Iberica, Almería, Spain) with TYLCD resistance based on the *Ty-1* resistance gene [34]. Plants inoculated with *A. tumefaciens* carrying the empty vector were used as the mock-inoculated control.

Agroinoculated plants were analyzed by squash blot hybridization using the probe specific to ToLCNDV DNA-A (see above) that also recognizes the Indian isolate. Symptom severity was evaluated at 15, 21 and 28 days post-inoculation (dpi) using a 0–5 arbitrary scale, where 0 was assigned to asymptomatic plants and 5 was assigned to the most severe symptoms observed. Infected tomato plants were maintained up to 60 dpi for symptom observation. Total DNA extracts obtained at 45 dpi from young leaves of agroinoculated plants negative by molecular hybridization were analyzed by PCR using the primers pair MA2059 (5′-AGCACAGCCACGGTGAAGAAC-3′) and MA2060 (5′-TTTCATCCTTCGACAGAGTTC-3′) for DNA-A and MA2061 (5′-AATACACGCGTAAGGAAATATGT-3′) and MA2062 (5′-AGTCATGGGCTAGCAGATCG-3′) for DNA-B [19].

**Figure 1:**
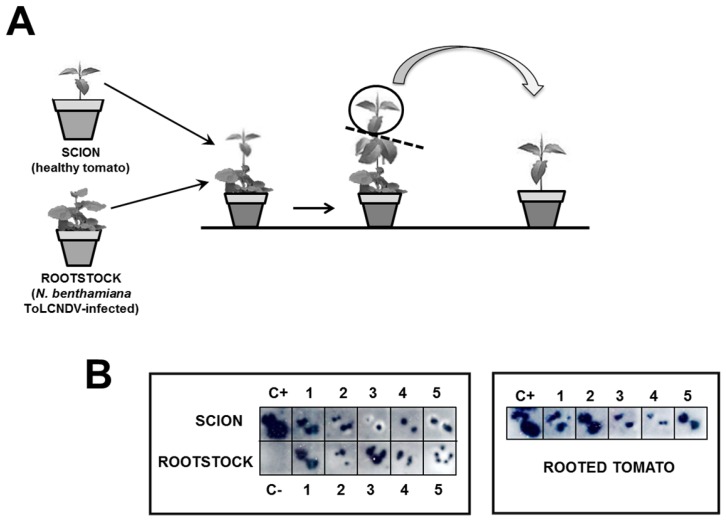
Graft assay. (**A**) Schematic representation of graft assay of healthy tomato cultivar Moneymaker scions on *Nicotiana benthamiana* rootstocks infected with the tomato leaf curl New Delhi virus (ToLCNDV) isolate from Spain (ToLCNDV-[ES-Alm-661-Sq-13]) followed by rooting detached apical tissues of infected tomato plants; (**B**) molecular hybridization of squash blots of cross-sections of petioles of young leaves of tomato scions performed on positively-charged nylon membranes at 90 days post grafting and at 30 days post rooting of apical tissues.

Grafting transmission experiments were performed in an insect-proof growth chamber (26 °C day and 18 °C night, 70% relative humidity, with a 16-h photoperiod at 250 μmol·s^−1^m^−2^ photosynthetically-active radiation) by using as scions healthy tomato cultivar Moneymaker plants and as rootstocks *N. benthamiana* plants previously infected with ToLCNDV-[ES-Alm-661-Sq-13] by agroinoculation using the DNA-A and DNA-B infectious clones. At 60 days post-grafting, the apical part of the tomato scion was analyzed for infection by squash blot hybridization and was then removed and rooted (Figure 1A). Plants were maintained in an insect-proof glasshouse (approximately 16-h day length at 24 °C and 18 °C at night, with light supplementation when needed) until their analysis by squash blot hybridization for virus presence and for symptoms assessment. New apical leaves were analyzed periodically up to 90 days after grafting in the case of tomato scions and *N. benthamiana* rootstocks and 30 days after rooting in the case of rooted tomatoes.

### 2.7. Whitefly Transmission

For whitefly transmission of ToLCNDV, adults of the *B. tabaci* Mediterranean (MED) species (formerly known as the Q biotype) and/or of *B. tabaci* Middle East–Asia Minor 1 (MEAM1) (formerly known as the B biotype) species [35] were used. Non-viruliferous whiteflies were reared on melon (Accession ANC 42, La Mayora-CSIC germplasm bank) in insect-proof cages. For virus acquisition, non-viruliferous whiteflies were given a 24-h acquisition access period (AAP) on systemically-infected young leaves of zucchini squash cultivar Milenio or tomato cultivar Moneymaker plants previously infected with ToLCNDV-[ES-Alm-661-Sq-13] by agroinoculation. ToLCNDV field-infected zucchini squash plants also were used as a virus source when needed. After the AAP, whiteflies were transferred to healthy test plants for a 48-h inoculation access period (IAP) (25–30 whiteflies per test plant in clip-on cages). After the IAP, insects were removed, and plants were treated with insecticide and maintained in insect proof-cages within an insect-proof glasshouse (see above) until analyzed and observed for symptom development. Inoculated plants were analyzed for ToLCNDV infection by squash blot hybridization of young non-inoculated tissues. In order to confirm the presence of ToLCNDV DNA-A and DNA-B components, total DNA was extracted from young non-inoculated leaves of test plants, and PCR amplification was conducted using the primers pairs MA1933/MA1934 and MA1935/MA1936. The products obtained were directly sequenced (Macrogen Inc., Seoul, Korea) when needed.

## 3. Results

### 3.1. Widespread Occurrence of ToLCNDV in Spanish Vegetable Growing Regions: Evidence for a Genetically-Uniform Population

A survey was conducted in commercial fields of the provinces of Málaga, Almería and Murcia (southern and southeastern Spain) for the detection of the presence of ToLCNDV. Plants with suspicious symptoms of ToLCNDV or begomovirus infection were collected in 2013, 2014 and 2015 from either zucchini squash, melon or tomato commercial crops in the major vegetable growing regions of Málaga, Almería and Murcia. Prevalences greater than 50% of symptomatic plants were observed in affected zucchini squash and melon crops surveyed, whereas in tomato, only TYLCD-like symptoms were observed. As can be seen in Table 1, high prevalence of ToLCNDV infection, close to 100%, was detected among symptomatic zucchini squash or melon plants collected in every surveyed field of Málaga, Almería and Murcia, suggesting a widespread occurrence of this virus in the sampled area. In contrast, although the ability of this virus to infect tomato plants has been reported in Spain [19], none of the plants collected from tomato crops growing in the vicinity of heavily infected zucchini squash crops exhibited ToLCNDV infection based on squash blot hybridization. All of the symptomatic tomato samples collected tested positive for probes specific to TYLCD-associated viruses.

**Figure 2:**
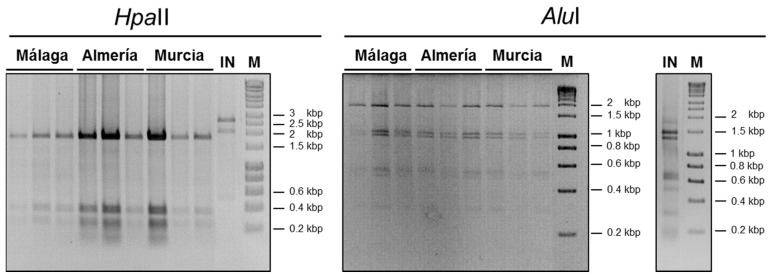
Restriction fragment length polymorphism (RFLP) analysis. Restriction fragment length polymorphism (RFLP) analysis of tomato leaf curl New Delhi virus (ToLCNDV)-infected samples from zucchini squash and melon plants collected in Málaga, Almería and Murcia provinces, performed by digestion of rolling circle amplification (RCA) products with the restriction enzymes *Hpa*II and *Alu*I, revealed on a 1.5% agarose gel, with three representative isolates per province shown. Equivalent RFLP analysis for a zucchini squash sample infected with a ToLCNDV from India (ToLCNDV-[India-New Delhi-Severe-1992], DNA-A and DNA-B GenBank Accession Numbers U15015 and U15017, respectively; labelled as IN) was included; M: 1-kb DNA ladder marker.

A total of 57 ToLCNDV isolates derived from the zucchini squash and melon samples that tested positive from Málaga, Almería or Murcia (Table 1) were analyzed by *Hpa*II and *Alu*I digestion of RCA amplification products. A characteristic and uniform RFLP pattern was obtained in all of the samples analyzed, consistent with an in silico cut of the DNA-A + DNA-B of the ToLCNDV genome of isolates reported in Spain. This pattern strongly differs from that obtained for samples of plants infected with a ToLCNDV isolate from India (Figure 2). These results suggested that the population of ToLCNDV analyzed is quite uniform, compatible with a founder effect in a recent introduction.

### 3.2. Genetic Relationships of ToLCNDV Isolates: A Complex Group with a New Strain of Recombinant Nature Emerging in Spain

Six complete genome sequences are available for ToLCNDV isolates present in the western Mediterranean Basin, all of them from Spain: ToLCNDV-[ES-Alm-661-Sq-13], ToLCNDV-[ES-MU-8.1-Sq-12], ToLCNDV-[ES-MU-11.1-Sq-12], ToLCNDV-[ES-Alm-Zucchini-13], ToLCNDV-[ES-Alm-TomA4-14] and ToLCNDV-[ES-Alm-TomA5-14] (Supplementary Materials Table S1) (DNA-A GenBank KF749223, KF749224, KF749225, KF891468, KM977733,and KT175406, respectively; DNA-B GenBank KF749226, KF749227, KF749228, KF891467, KM977734 and KT175407, respectively). The nucleotide sequence of the DNA-A components of these isolates was compared to full-length DNA-A sequences available in databases for ToLCNDV isolates. A total of 126 DNA-A full-length sequences is available for isolates mostly from Asia (mainly from India and Pakistan, but also from Bangladesh, Thailand, Taiwan, Indonesia and Iran) (Supplementary Materials Table S1). Based on pairwise nucleotide sequence comparisons (Supplementary Materials Figure S1) and criteria for species/strain sequence demarcation currently accepted for begomoviruses (≥91% and ≥94% nucleotide sequence identity for species and strain demarcation, respectively) [3], it was shown that most of the ToLCNDV isolates from the Asian locations were in a single group of closely-related DNA-A sequences (≥94% identity) despite the high diversity of geography, year of sampling and host species origin. In contrast, the DNA-As of the six isolates reported from Spain fell into a group of closely-related isolates (greater than 99% nucleotide sequence identity) with less than 94% homology to DNA-As of ToLCNDV reported so far (Supplementary Materials Figure S1). Therefore, they were suggested to constitute a new strain of this virus. Furthermore, a number of ToLCNDV isolates from India and Pakistan (highlighted with asterisks in Supplementary Materials Figure S1 and Table S1) was observed, which differ from the reported ones at the strain threshold level. These included the isolate with DNA-A GenBanK KC465466 from India that was classified by Brown et al. [3] as a new species, named ToLCNDV3-[IN-Bij-Chi-12], but it exhibited 91% nucleotide identity with ToLCNDV-[PK-Lah-Sn-04] (GenBank AM849548). Therefore, following species/strain demarcation criteria for the genus *Begomovirus* [3], this isolate belongs to ToLCNDV.

The SDT analysis showed that the DNA-As of two isolates that were submitted to GenBank as ToLCNDV (ToLCNDV-[IN-Har-03], GenBank FJ561298, and ToLCNDV-[BD-cuc-06], GenBank EF450316), exhibited DNA-A identity percentages below 91% of that of any ToLCNDV reported so far, with maximum identity percentages of 90.00% and 90.40%, respectively (Supplementary Materials Figure S1). Therefore, based on the species demarcation criteria for the genus *Begomovirus* [3], these isolates do not belong to the ToLCNDV species. Similarly, as reported previously by Brown et al. [3], isolates ToLCNDV2-[IN-IANDS1-11] (GenBank JQ897969) and ToLCNDV4-[IN-Jun-TC306-11] (GenBank KF551592), which exhibited nucleotide identities below 91% with any ToLCNDV, also correspond to different begomovirus species.

**Figure 3:**
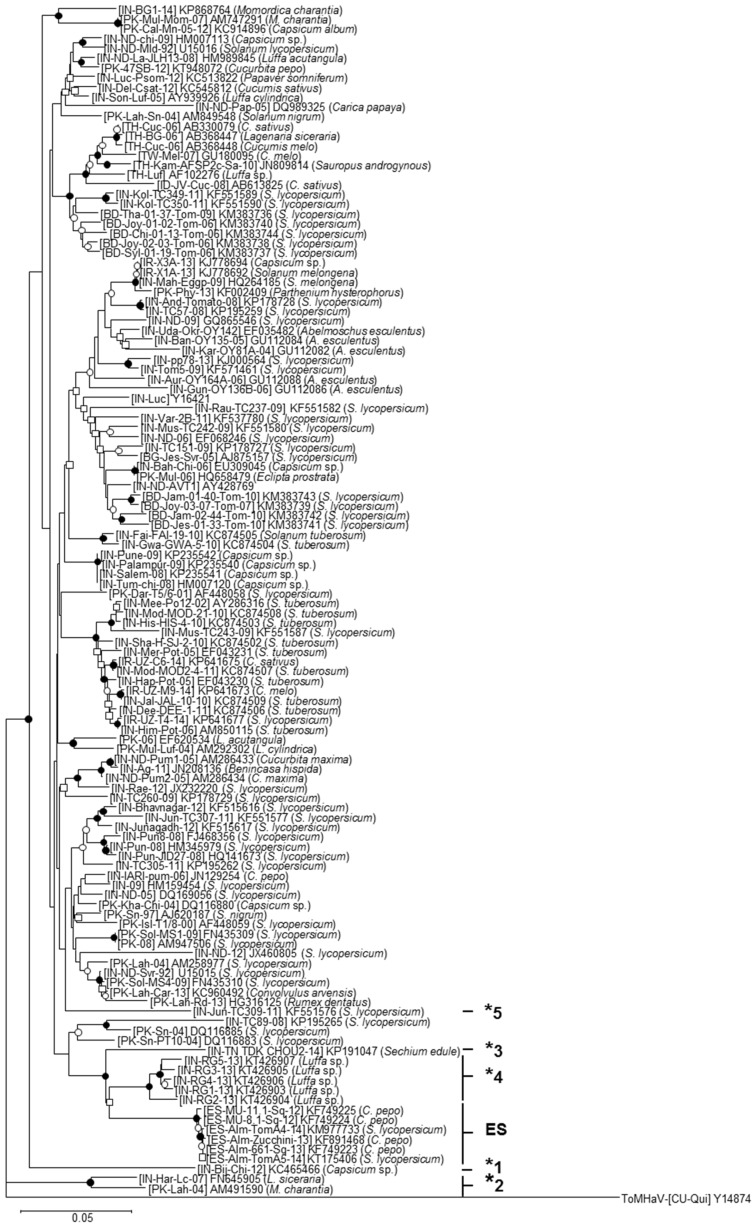
Phylogenetic relationships among DNA-A sequences of ToLCNDV isolates. Phylogenetic relationships among the full-length DNA-A sequences of 126 tomato leaf curl New Delhi virus (ToLCNDV) isolates available in the NCBI-GenBank and International Committee on Taxonomy of Viruses 2016 databases as of 3 May 2016. The tree was constructed using the neighbor-joining method with the MEGA 5.05 software program [29]. Bootstrap (1000 replicates) values are expressed as percentages, and only the nodes with values greater than 50% are labeled as follows: nodes supported in >90% and >70% of bootstrap replicates are marked with filled and open circles, respectively, and those with values greater than 50% are marked with open squares. Isolates that potentially correspond to different strains are highlighted with asterisks (“ES” for the Spanish isolates); the same number/letters indicate isolates within the same relationship group according to Supplementary Materials Figure S1. The scale bar indicates 0.05 nucleotide substitutions per site. Virus species, GenBank accession number and plant host are indicated for each isolate included in the analysis. The sequence of the DNA-A of isolates [CU-Qui] of the bipartite begomovirus species *Tomato mosaic Havana virus isolate* (ToMHaV) (GenBank Accession Number Y14874) was included as an outgroup.

As shown in Figure 3, the phylogenetic relationships of the DNA-A sequences of ToLCNDV showed that the isolates from Spain were grouped in a separate clade. No other evident topological group of the ToLCNDV population was observed associated with plant host, year of sampling or geographical location. Isolates ToLCNDV-[IN-Har-Lc-07] and ToLCNDV-[PK-Lah-04] constituted a topological group in the basal part of the tree robustly separated from all other ToLCNDVs.

**Figure 4:**
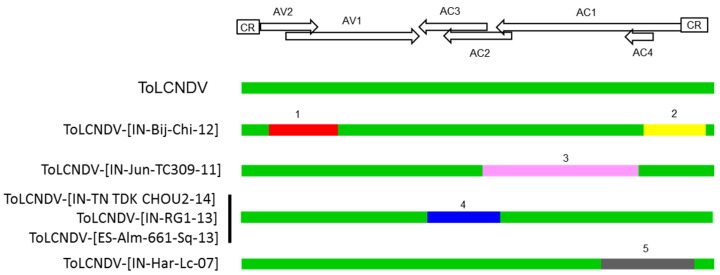
Putative recombination events of the isolates of ToLCNDV. Schematic representation of the putative recombination events present in the DNA-A of isolates of tomato leaf curl New Delhi virus (ToLCNDV) that differ from the reported ones at the strain threshold level detected using the Sequence Demarcation Tool (SDT) analysis (highlighted with asterisks in Figure 3) compared to ToLCNDV standard isolates and deduced based on the results from the recombination detection software program (RDP4; Version 4.0) [31]. Whereas the green color indicates ToLCNDV-derived sequences, all other colors indicate sequences derived from non-ToLCNDV sources, with recombination events numbered according to Table 2.

We analyzed the possible recombination origin of isolates that differ at the strain threshold level in the SDT analysis using the RDP4 program. We found support for the recombination origin of their DNA-As, with five putative recombination events (detectable by three or more different analysis methods) (Table 2 and Figure 4). Isolates [IN-TN TDK CHOU2-14], [IN-RG1-13] and [ES-Alm-661-Sq-13] exhibited DNA-As with similar recombination structures that subsequently seemed to have diverged from each other by point mutations. Therefore, we can conclude that ToLCNDV exhibits a complex population in which recombination and point mutations seem to be driving virus evolution.

### 3.3. The ToLCNDV Isolates of the Novel Strain Emerging in Spain Are Infectious in Cucurbits, but Poorly Adapted to Tomato

To evaluate the biological properties of the isolates of the ToLCNDV strain emerging in Spain, infectious clones were prepared for the DNA-A and DNA-B components of the Spanish isolate ToLCNDV-[ES-Alm-661-Sq-13] (ND-AL-661-2.0-A and ND-AL-661-2.0-B, respectively). *N. benthamiana* and zucchini squash plants agroinoculated with these clones resulted in 100% infection success (10 plants systemically infected of 10 plants inoculated). Inoculated zucchini squash plants exhibited symptoms of mosaic and curling in apical leaves similar to those observed in field-infected zucchini squash plants. Moreover, when using agroinoculated zucchini squash-infected plants as an inoculum source for *B. tabaci* acquisition of ToLCNDV, the virus was efficiently acquired and transmitted to healthy zucchini squash plants (nine plants systemically infected out of 10 inoculated, with 25 whiteflies used per test plant). *B. tabaci*-inoculated plants exhibited the same symptoms described above. The presence of the DNA-A and DNA-B of the inoculated virus in newly-emerged non-inoculated apical leaves of infected test plants was confirmed by PCR amplification and direct sequencing of the amplified fragment using the primer pairs MA1933/MA1934 and MA1935/MA1936. Therefore, full infectious and biologically-active clones of DNA-A and DNA-B of ToLCNDV-[ES-Alm-661-Sq-13] were obtained that induced disease symptoms in zucchini squash that were indistinguishable from those observed in the field.

Once the infectivity of the cloned DNA-A and DNA-B of ToLCNDV-[ES-Alm-661-Sq-13] was confirmed with agroinoculation, the host range of this isolate was studied. Plants of cultivated hosts were tested, composed of species of the *Cucurbitaceae* (zucchini squash, melon, watermelon and cucumber), *Fabaceae* (common bean) and *Solanaceae* (tomato and pepper) families. Furthermore, plants of a wild species (*S. nigrum*, belonging to the *Solanaceae*) frequently associated with those cultivated species were tested. In addition, plants of several experimental hosts (*N. benthamiana*, *N. tabacum* cultivar Xanthi and *N. glutinosa* of the family *Solanaceae*) also were tested. The results summarized in Table 3 showed the ability of this virus to infect the cucurbit hosts tested. A similar ability was also observed for the *Nicotiana* species tested. All infected plants exhibited clear infection symptoms (mosaic and leaf curling in leaves) at 30 dpi. In contrast, no systemic infection was achieved for plants of common bean or *S. nigrum*. Similar negative infection results were obtained for pepper and for all three *A. tumefaciens* strains used (results for C58C1 shown in Table 3). For tomato (cultivar Moneymaker), only one out of 20 plants inoculated in two replicated experiments exhibited systemic infection. This one plant was positive for molecular hybridization, but did not exhibit symptoms even at 60 dpi. Control inoculations performed with the Indian isolate of ToLCNDV (ToLCNDV-[IN-ND-Svr-92]) showed that this virus was highly infectious in tomato, zucchini squash and *N. benthamiana* with a 100% infection rate and severe symptoms induced in all of the infected plants at 30 dpi. Virus (DNA-A and DNA-B) presence in hybridization-positive plants was confirmed by PCR. All plants that tested negative for hybridization also were negative for PCR.

**Figure 5:**
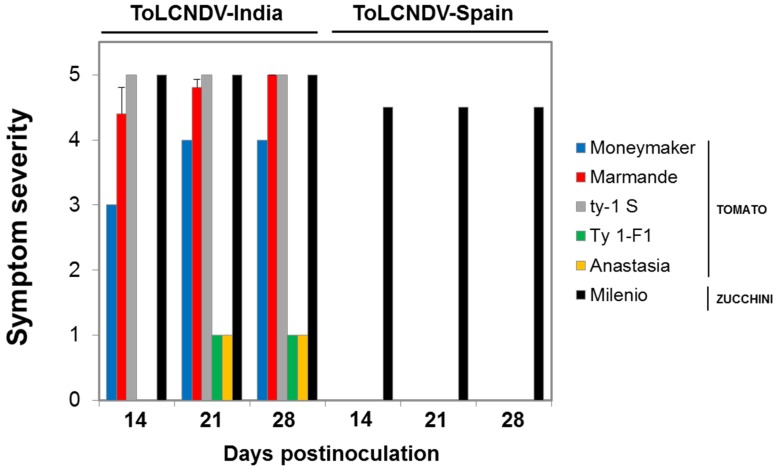
Severity of ToLCNDV infection in tomato and zucchini squash plants. Evolution of symptom severity (0, no symptoms; 5, maximum symptom severity observed for the Indian isolate) in tomato and zucchini squash plants agroinoculated with tomato leaf curl New Delhi virus (ToLCNDV) isolates from Spain (ToLCNDV-[ES-Alm-661-Sq-13]) and India (ToLCNDV-[IN-ND-Svr-92]) (Assay 1 in Table 4); values are the mean ± standard error for infected plants of 10 tomato and zucchini squash plants inoculated.

As the presence of ToLCNDV infections was reported recently in tomato plants from commercial crops in Spain [19], further evaluation of additional tomato cultivars and genotypes was conducted by agroinoculation and/or by *B. tabaci*-mediated inoculation either using the cloned or a field-collected ToLCNDV isolate. The infection results obtained are summarized in Table 4. These results again showed a poor ability of the Spanish isolate ToLCNDV-[ES-Alm-661-Sq-13] to infect tomato even by *B. tabaci*-mediated inoculations. In the latter case, similar poor infection results were obtained for the cloned isolate either acquired from zucchini squash source plants or from a tomato cultivar Moneymaker plant that was infected by agroinoculation and the infection maintained with this isolate for more than six months. Therefore, no selection of a tomato-adapted variant with time seemed to occur in this infected tomato plant. Symptoms observed in tomato and zucchini-infected plants were similar among inoculation assays (represented in Figure 5 for a number of genotypes of the agroinoculation Assay 1). Equivalent to the previous inoculation study (Table 3), no symptoms of ToLCNDV infection with the Spanish isolate were observed in infected tomato plants even at 60 dpi, whereas characteristic and severe symptoms of infection were observed in infected zucchini squash plants.

It should be highlighted that similar poor infection ability and absence of symptoms in tomato was observed for a ToLCNDV isolate collected from a field-infected zucchini squash plant either with the MED or the MEAM1 *B. tabaci* types for transmission, whereas highly efficient transmission was achieved in zucchini squash. The latter indicated similar behavior for the cloned and the field-collected ToLCNDV isolate, suggesting that an isolate with poor infectivity in tomato was not selected during the cloning process. In contrast, highly efficient infections were obtained either for tomato or zucchini squash with the Indian ToLCNDV isolate used. Interestingly, in the latter case, effective resistance to ToLCNDV virus infection and reduced symptom severity was observed in the tomato cultivar Anastasia, a commercial tomato resistant to TYLCD (based on the *Ty-1* resistance gene) [35]. A substantially reduced rate of infection was observed at 28 dpi compared to 21 dpi. As shown in Figure 5, the Indian ToLNCDV isolate induced severe symptoms of infection either in tomato or zucchini squash plants infected, with the exception of the TYLCD-resistant *Ty-1*-F_1_ line and cultivar Anastasia tomatoes in which very mild symptoms of infection were observed in infected plants. Therefore, the *Ty-1* could be an effective gene for the management of ToLCNDV, as previously reported in India.

Further studies on the ability of the Spanish isolate ToLCNDV-[ES-Alm-661-Sq-13] to infect tomato plants were conducted based on grafting experiments. As shown in Figure 1B for healthy tomato cultivar Moneymaker plants grafted onto ToLCNDV-infected *N. benthamiana* rootstocks, newly-grown leaves of all grafted tomato plants exhibited clear ToLCNDV infection at 21–90 days post grafting (exemplified in Figure 1B for 21 days post grafting). Furthermore, all rooted cuttings from apical tissues of the infected tomato scions remained infected at 21 and 30 days after rooting (shown in Figure 1B for 30 days after rooting). Therefore, although based on a single experiment, this suggests that continuous ToLCNDV virus inoculation facilitates infection of tomato by this virus. Nevertheless, even in these conditions, none of the tomato plants that tested positive for ToLCNDV-[ES-Alm-661-Sq-13] infection exhibited symptoms.

## 4. Discussion

Continuous emergence of begomoviruses has occurred throughout the past 20 years leading to global disease outbreaks mainly driven by the emergence of their vector, the whitefly *B. tabaci* [36]. Here, we showed that the begomovirus ToLCNDV exhibits a complex population with isolates reported in a wide range of plant host species (at least for a begomovirus) and countries mainly from the Indian subcontinent and other Asian countries. Except for the group of isolates recently detected in Spain, no clear geographical, year of sampling or plant host species phylogenetic group was observed. However, an analysis based on pairwise comparisons of DNA-A nucleotide sequences of ToLCNDV isolates reported so far and following ICTV demarcation criteria for begomoviruses [3] suggested the presence of a novel strain spreading in the western Mediterranean Basin. Furthermore, several additional strains might be present in the population, that await further study before names are given [37]. The presence of three differentiated strains within the ToLCNDV complex already has been suggested by Jyothsna et al. on the basis of nucleotide sequence identity [38] after the analysis of a more limited number of sequences. A predominant and widely-distributed group of closely-related ToLCNDV isolates was found present in countries of the Indian subcontinent, Southeast Asia and the Middle East. The study of sequences that diverged from this major group suggested that recombination seemed to be a major force driving the evolution of ToLCNDV as already shown for many other geminiviruses [39,40]. It should be highlighted that as observed in other begomovirus systems [41,42], some of these divergent isolates can emerge, causing epidemics. This might be the case of ToLCNDV isolates detected in Spain, which are closely related genetically and exhibit a recombinant origin of their DNA-A. This group of isolates differed at the strain level, and the name “Spain (ES)” was proposed for the strain [16,43]. Isolates closely associated with this strain also have been reported to cause severe epidemics in cucurbits in Tunisia and Italy [17,19,20,21], suggesting the emergence of the ES strain in the western Mediterranean Basin. Here, we demonstrated that in the ToLCNDV Spanish epidemics, the population of ToLCNDV isolates involved was quite uniform genetically, probably the result of a founder effect associated with a population bottleneck during the transmission of this virus to a new area [44,45,46].

An infectious clone was obtained for one of the ToLCNDV isolates collected from zucchini squash in Spain. Biological studies indicated that, similarly to a field-collected isolate, this cloned isolate exhibited a good ability to infect cucurbit plants, as shown previously for another Spanish isolate [18]. However, a poor adaptation to infect tomatoes was observed. This contrasted with the efficient and severe infections caused in tomato by an Indian isolate of ToLCNDV used as a control that also induced severe infections in cucurbits. It is known that even limited genetic variations can determine changes in the host range or aggressiveness of a virus [41,47,48]. Genomic comparisons between the Spanish and the Indian ToLCNDV isolates showed the existence of four regions in the DNA-A with low nucleotide sequence identity and showed that, in general, most regions of DNA-B are below 90% identity (Supplementary Materials Figure S2). As infectious clones are available for both viruses, studies to understand virus determinants associated with the ability to infect tomato are feasible.

It is worth mentioning that Ruiz et al. [19] reported ToLCNDV infections in commercial tomatoes grown in Almería, Spain. Furthermore, these same authors recently obtained an infectious clone for one of the isolates collected from zucchini squash in Almería (isolate ToLCNDV-[ES-Alm-Zucchini-13]) and demonstrated that it was able to infect readily and induce symptoms in tomato [43]. Sequence comparison indicated that few nucleotide differences scattered in the genome are observed between the isolate cloned by Ruiz et al. [43] and the one for which an infectious clone was obtained here. Based on the infectious clones available, further research then might help to localize viral genetic determinants associated with infection and symptom induction in tomato. Taking into account the previous observations, surveillance and control of ToLCNDV in the epidemics occurring in Spain and other affected places of the Mediterranean Basin will be essential to contain and control the possible spread of this virus to other cultivated species, such as tomato. Nevertheless, if efficient spread to tomato occurs, here we showed that the *Ty-1* gene already deployed for the control TYLCD-associated viruses [49] seems to provide resistance to ToLCNDV, even for the isolate from India that causes severe infections in susceptible tomatoes. Moreover, other gene combinations have been reported to provide effective resistance to ToLCNDV infections in tomato [50,51].

Description of the presence of ToLCNDV in Spain was the first report of a bipartite begomovirus causing epidemics in the western Mediterranean Basin where tomatoes have been severely affected by several monopartite begomoviruses associated with TYLCD since the early 1990s [52]. Cucurbits are good hosts for ToLCNDV and also can host TYLCD-associated viruses (e.g., [53]). Similarly, ToLCNDV might infect tomato where TYLCD-associated viruses are frequent. Therefore, the possible occurrence of mixed infection between ToLCNDV and TYLCD-associated begomoviruses either in tomato or cucurbits is a cause of concern because begomoviruses have a great potential for recombination, which can have dramatic pathogenic consequences. In fact, the possible recombination between monopartite and bipartite begomoviruses and involvement in the evolution and emergence of new and more pathogenic virus species already has been reported [54,55,56]. Moreover, the exchange of DNA components (pseudo-recombination) might occur between begomoviruses, which can determine changes in the aggressiveness and host range. The latter was shown for example for pseudo-recombination between isolates of the bipartite begomoviruses ToLCNDV and tomato leaf curl Gujarat virus that can result in extremely severe infections in tomato [15] or determine a resistance break in chili pepper plants [57]. Viable pseudo-recombination between monopartite and bipartite begomoviruses has been reported, with evidence for acquisition of a DNA-B component by a monopartite begomovirus (e.g., [58]). As DNA-B contains genes encoding the movement functions [59], its acquisition can determine novel host range abilities. Furthermore, synergistic interactions might occur in mixed infections [57], which eventually can lead to increased damage or even overcoming of resistance [57,60,61].

Taken together, our results support that outbreaks of ToLCNDV in the western Mediterranean Basin are associated with a founder effect, with isolates of a novel recombinant strain of ToLCNDV causing severe damage to cucurbit crops. Concerns exist associated with their progressive spread and threat to additional vegetable crops if mixed infections occur with begomoviruses already present in the region that can determine synergistic interactions and/or a novel source of genetic variation by recombination.

## Figures and Tables

**Table 1:** Prevalence of tomato leaf curl New Delhi virus (ToLCNDV) in plants collected from commercial fields of zucchini squash, melon and tomato of Málaga, Almería and Murcia provinces (southern and southeastern Spain) during a survey conducted from 2013–2015.

Year	Province	No. of ToLCNDV Positive Samples/No. of Total Analyzed Samples/No. of Fields Surveyed ^a^		
		Zucchini Squash	Melon	Tomato
2013	Málaga	6/6/2	-	-
	Almería	20/20/3	-	-
	Murcia	4/4/2	-	-
2014	Málaga	8/8/1	1/1/1	-
	Almería	24/26/3	2/2/1	0/3/2
	Murcia	-	-	0/10/1
2015	Málaga	10/10/1	6/6/1	0/81/3
	Almería	-	-	0/17/2
	Murcia	-	-	0/1/1
**Total**		72/74/12	9/9/3	0/112/9

^a^ Samples were analyzed by hybridization of squash blots of cross-sections of petioles of young leaves made on positively-charged nylon membranes. For detection, a digoxigenin-labelled DNA probe specific to ToLCNDV was used.

**Table 2:** Major recombination events detected among isolates of ToLCNDV that differ with reported ones at the strain threshold level noticed using the Sequence Demarcation Tool (SDT) analysis (highlighted with asterisks in Figure 3) and isolates of closely-related begomoviruses putatively involved in the recombination events.

Event Number	Recombinant Sequence	Putative Non-ToLCNDV Parent	Putative ToLCNDV Parent	Breakpoints ^a^		Methods ^b^	*p*-Value ^c^
				Begin	End		
1	ToLCNDV-[IN-Bij-Chi-12]	ChiLCPKV-PK[PK-Kha-04]	ToLCNDV-[BG-Jes-Svr-05]	146	547	RGBMCST	8642 × 10^−16^
2	ToLCNDV-[IN-Bij-Chi-12]	Unknown ^d^	ToLCNDV-[IN-ND-Pap-05]	2328	2743	RGBMCST	4332 × 10^−15^
3	ToLCNDV-[IN-Jun-TC309-11]	ToLCPalV-[IN-Rau-TC238-09]	ToLCNDV-[IN-ND-Pap-05]	1397	2301	RGBMCST	4722 × 10^−7^
4	ToLCNDV-[IN-TN TDK CHOU2-14]	ToLCPalV-[IR-Jir-T69P-Cuc-08]	ToLCNDV-[IN-Har-Lc-07]	1055	1532	RMC	8702 × 10^−5^
4	ToLCNDV-[IN-RG1-13]	ToLCPalV-[IR-Jir-T69P-Cuc-08]	ToLCNDV-[IN-Har-Lc-07]	1054	1524	RMC	8702 × 10^−5^
5	ToLCNDV-[IN-Har-Lc-07]	Unknown	ToLCNDV-[IN-ND-Pap-05]	2063	2661	RGBMCST	6679 × 10^−11^
4	ToLCNDV-[ES-Alm-661-Sq-13]	ToLCPalV-[IR-Jir-T69P-Cuc-08]	ToLCNDV-[IN-Har-Lc-07]	1054	1531	RMC	8702 × 10^−5^

^a^ Positions in the recombinant sequence; ^b^ only events detected with three or more methods were considered credible evidence of recombination. R = RDP, G = GENECONV, B = BootScan, M = MaxChi, C = Chimaera, S = SiScan, T = 3Seq; ^c^ the reported *p*-value is the highest obtained for that region; ^d^ unknown; only one parent and a recombinant need be in the alignment for a recombination event to be detectable; the putative non-ToLCNDV parent is unknown.

**Table 3:** Inoculation of the Spanish isolate [ES-Alm-661-Sq-13] of ToLCNDV in plants of a range of cultivated, wild and experimental host species by means of *Agrobacterium tumefaciens*-mediated inoculation; the Indian isolate ToLCNDV-[IN-ND-Svr-92] (GenBank Accession Numbers U15015 and U15017 for DNA-A and DNA-B, respectively) was included as a control.

Host Type	Family	Species	Cultivar/Genotype	Infected Plants/Total Inoculated Plants ^a^		
				Spanish Isolate ^b^		Indian Isolate ^c^
				Assay 1	Assay 2	Assay 1
**Cultivated host**	*Cucurbitaceae*	Zucchini (*Cucurbita pepo*)	Milenio	5/5	5/5	5/5
			Afrodita	5/5	5/5	-
		Melon (*Cucumis melo*)	C-278	3/5	0/5	-
		Watermelon (*Citrullus lanatus*)	SV80287	4/5	0/5	-
		Cucumber (*Cucumis sativus*)	Pacer	5/5	0/5	-
			Bell Puig	4/5	2/5	-
	*Fabaceae*	Bean (*Phaseolus vulgaris*)	Donna	0/5	0/5	-
	*Solanaceae*	Tomato (*Solanum lycopersicum*)	Moneymaker	0/10	1/10	9/10
		Pepper (*Capsicum annuum*)	Jalapeño	0/5	0/5	-
			C. Wonder	0/5	0/5	-
**Experimental host**	*Solanaceae*	*Nicotiana benthamiana*		5/5	5/5	5/5
		*Nicotiana tabacum*	Xanthi	3/5	1/5	-
		*Nicotiana glutinosa*		5/5	5/5	-
**Wild host**	*Solanaceae*	*Solanum nigrum*		0/5	0/5	-

^a^ Inoculated plants were analyzed at 30 days post-inoculation for the presence of disease symptoms by visual observation and for the presence of viruses in newly-emerged young leaves by tissue-blot hybridization. All infected plants developed symptoms of infection (mosaic and leaf curling), except for the tomato plants inoculated with the Spanish isolate, which remained asymptomatic. All plants that tested negative for squash blot hybridization were negative for PCR using the primer pair MA2059/MA2060; ^b^ [ES-Alm-661-Sq-13]; ^c^ [IN-ND-Svr-92]; “-”: not tested.

**Table 4:** Infectivity in plants of different cultivars/genotypes of tomato of the Spanish isolate [ES-Alm-661-Sq-13] of ToLCNDV or a field-collected ToLCNDV isolate inoculated by *Agrobacterium tumefaciens*-mediated and/or *Bemisia tabaci*-mediated (Mediterranean, MED, and Middle East–Asia Minor 1, MEAM1, species used) inoculation. The Indian isolate ToLCNDV-[IN-ND-Svr-92] was included as a control in agroinoculation experiments, and zucchini squash plants were included as a susceptible control. Inoculated plants were analyzed at 28 days post-inoculation for the presence of viruses in newly-emerged young leaves by squash blot hybridization.

Crop/Cultivar or Line	Agroinoculation				Whitefly Inoculation			
					Zucchini Source ^a^			Tomato Source ^a^
	Spanish Isolate ^b^			Indian Isolate ^d^	Spanish Isolate ^b^	Spanish Field Inoculum ^c^		Spanish Isolate ^b^
					MED Species	MED Species	MEAM1 Species	MED Species
	Infected Plants/Total Plants Inoculated			Infected Plants/Total Plants Inoculated	Infected Plants/Total Plants Inoculated	Infected Plants/Total Plants Inoculated	Infected Plants/Total Plants Inoculated	Infected Plants/Total Plants Inoculated
	Assay 1	Assay 2	Assay 3	Assay 1				
**TOMATO**								
Moneymaker	1/10	-	0/10	10/10	1/10	0/10	0/10	0/10
Fortuna C	2/10	1/10	-	7/10	-	-	-	-
Gardener’s Delight	0/10	0/10	-	10/10	-	-	-	-
Rondeño	2/10	2/10	1/10	10/10	0/10	0/10	0/10	0/10
Marmande	4/10	1/10	-	10/10	0/10	0/10	0/10	0/10
*Ty-1* S	1/10	0/10	-	10/10	0/10	1/10	0/10	-
*Ty-1* F_1_	0/10	0/10	-	10/10 ^e^	0/10	0/10	0/10	-
Anastasia	0/10	0/10	-	2/10 ^e,f^	-	-	-	-
**ZUCCHINI**								-
Milenio	5/5	5/5	5/5	5/5	5/5	5/5	5/5	5/5

^a^ Host species used as ToLCNDV inoculum source; ^b^ [ES-Alm-661-Sq-13]; ^c^ young leaves collected from a zucchini squash plant infected with ToLCNDV from a commercial field were used as a source of virus inoculum; ^d^ [IN-ND-Svr-92]; ^e^ although squash blot is not a quantitative technique, low hybridization signals were observed; ^f^ all plants tested were positive at 21 dpi; “-”: not tested.

## Supplementary Materials

The following are available online at www.mdpi.com/1999-4915/8/11/307/s1, Figure S1: Two-dimensional identity matrix between full-length tomato leaf curl New Delhi virus (ToLCNDV) DNA-A sequences, Figure S2: Plot similarity diagrams comparing DNA-A and DNA-B nucleotide sequences of ToLCNDV isolates, Table S1: Name, acronym and accession number of the DNA-A component of ToLCNDV isolates used in this study.
