# Supplementary material for: A Novel Strain of Tomato Leaf Curl New Delhi Virus Has Spread to the Mediterranean Basin
Source: Viruses. 2016 Nov 11;8(11):307. doi: 10.3390/v8110307 (PMC5127021; doi:10.3390/v8110307)
Supplement: Supplementary file 1 [file viruses-08-00307-s001.zip › viruses-140137-Supplementary Materials/viruses-140137-supplementary Table S1 and Figure S2.pdf]

Supplementary Materials: A Novel Strain of Tomato Leaf Curl New Delhi Virus Has Spread to the Mediterranean Basin

Isabel M. Fortes, Sonia Sánchez-Campos, Elvira Fiallo-Olivé, Juan A. Díaz-Pendón, Jesús Navas-Castillo and Enrique Moriones

Table S1. Name, acronym and accession numbers of DNA-A component of tomato leaf curl New Delhi virus isolates used in this study. In the "Strain" column, isolates that potentially correspond to different strains are highligthed with asterisks ("ES" for the Spanish isolates); same number/letters indicates isolates within the same relationship group according to Supplementary Figure S1; "Other species" indicates sequences that do not correspond to *Tomato leaf curl New Delhi virus* species according to ICTV criteria.

Tomato Leaf Curl New Delhi virus isolate	Acronym	DNA-A GenBank Accession Number (DNA-A)	Strain
Tomato leaf curl New Delhi virus - [India-New Delhi-Severe-1992]	ToLCNDV-[IN-ND-Svr-92]	U15015	
Tomato leaf curl New Delhi virus - [India-New Delhi-Mild-1992]	ToLCNDV-[IN-ND-Mld-92]	U15016	
Tomato leaf curl New Delhi virus - [India-Meerut-Potato 12-2002]	ToLCNDV-[IN-Mee-Po12-02]	AY286316	
Tomato leaf curl New Delhi virus - [India-Haryana-2003]	ToLCNDV-[IN-Har-03]	FJ561298	Other specie
Tomato leaf curl New Delhi virus - [India-Karnal-OY81A-2004]	ToLCNDV-[IN-Kar-OY81A-04]	GU112082	
Tomato leaf curl New Delhi virus - [India-New Delhi-2005]	ToLCNDV-[IN-ND-05]	DQ169056	
Tomato leaf curl New Delhi virus - [India-New Delhi-Pumpkin 1-2005]	ToLCNDV-[IN-ND-Pum1-05]	AM286433	
Tomato leaf curl New Delhi virus - [India-New Delhi-Pumpkin 2-2005]	ToLCNDV-[IN-ND-Pum2-05]	AM286434	
Tomato leaf curl New Delhi virus - [India-Happur-Potato-2005]	ToLCNDV-[IN-Hap-Pot-05]	EF043230	
Tomato leaf curl New Delhi virus - [India-Meerut-Potato-2005]	ToLCNDV-[IN-Mer-Pot-05]	EF043231	
Tomato leaf curl New Delhi virus - [India-Bangalore-OY135-2005]	ToLCNDV-[IN-Ban-OY135-05]	GU112084	
Tomato leaf curl New Delhi virus - [India-Sonepat-Luffa-2005]	ToLCNDV-[IN-Son-Luf-05]	AY939926	
Tomato leaf curl New Delhi virus - [India-New Delhi-Papaya-2005]	ToLCNDV-[IN-ND-Pap-05]	DQ989325	
Tomato leaf curl New Delhi virus - [India-New Delhi-2006]	ToLCNDV-[IN-ND-06]	EF068246	
Tomato leaf curl New Delhi virus - [India-IARI-Pumpkin-2006]	ToLCNDV-[IN-IARI-pum-06]	JN129254	
Tomato leaf curl New Delhi virus - [India-Himachal-Potato-2006]	ToLCNDV-[IN-Him-Pot-06]	AM850115	

Table S1. *Cont.*

Tomato Leaf Curl New Delhi virus isolate	Acronym	DNA-A GenBank Accession Number (DNA-A)	Strain
Tomato leaf curl New Delhi virus - [India-Guntur-OY136B-2006]	ToLCNDV-[IN-Gun-OY136B-06]	GU112086	
Tomato leaf curl New Delhi virus - [India-Aurangabad-OY164A-2006]	ToLCNDV-[IN-Aur-OY164A-06]	GU112088	
Tomato leaf curl New Delhi virus - [India-Bahraich-Chilli-2006]	ToLCNDV-[IN-Bah-Chi-06]	EU309045	
Tomato leaf curl New Delhi virus - [India-Haryana-Lagenaria ciceraria-2007]	ToLCNDV-[IN-Har-Lc-07]	FN645905	*2
Tomato leaf curl New Delhi virus - [India-Tumkur-Chilli-2008]	ToLCNDV-[IN-Tum-chi-08]	HM007120	
Tomato leaf curl New Delhi virus - [India-New Delhi-Lufa acutangula-JLH13-2008]	ToLCNDV-[IN-ND-La-JLH13-08]	HM989845	
Tomato leaf curl New Delhi virus - [India-Pune-2008]	ToLCNDV-[IN-Pun-08]	HM345979	
Tomato leaf curl New Delhi virus - [India-Pune-JID27-2008]	ToLCNDV-[IN-Pun-JID27-08]	HQ141673	
Tomato leaf curl New Delhi virus - [India-Pune 8-2008]	ToLCNDV-[IN-Pun8-08]	FJ468356	
Tomato leaf curl New Delhi virus - [India-TC55-2008]	ToLCNDV-[IN-And-Tomato-08]	KP178728	
Tomato leaf curl New Delhi virus - [India-TC89-2008]	ToLCNDV-[IN-TC89-08]	KP195265	
Tomato leaf curl New Delhi virus - [India-TC57-2008]	ToLCNDV-[IN-TC57-08]	KP195259	
Tomato leaf curl New Delhi virus - [India-Salem-2008]	ToLCNDV-[IN-Salem-08]	KP235541	
Tomato leaf curl New Delhi virus - [India-Muskabad-TC242-2009]	ToLCNDV-[IN-Mus-TC242-09]	KF551580	
Tomato leaf curl New Delhi virus - [India-New Delhi-Chilli-2009]	ToLCNDV-[IN-ND-chi-09]	HM007113	
Tomato leaf curl New Delhi virus - [India-Tom5-2009]	ToLCNDV-[IN-Tom5-09]	KF571461	
Tomato leaf curl New Delhi virus - [India-2009]	ToLCNDV-[IN-09]	HM159454	
Tomato leaf curl New Delhi virus - [India-Maharastra-Eggplant-2009]	ToLCNDV-[IN-Mah-Eggp-09]	HQ264185	
Tomato leaf curl New Delhi virus - [India-Rauke-TC237-2009]	ToLCNDV-[IN-Rau-TC237-09]	KF551582	
Tomato leaf curl New Delhi virus - [India-New Delhi-2009]	ToLCNDV-[IN-ND-09]	GQ865546	
Tomato leaf curl New Delhi virus - [India-Muskabad-TC243-2009]	ToLCNDV-[IN-Mus-TC243-09]	KF551587	
Tomato leaf curl New Delhi virus - [India-TC151-2009]	ToLCNDV-[IN-TC151-09]	KP178727	
Tomato leaf curl New Delhi virus - [India-Palampur-2009]	ToLCNDV-[IN-Palampur-09]	KP235540	
Tomato leaf curl New Delhi virus - [India-Pune-2009]	ToLCNDV-[IN-Pune-09]	KP235542	
Tomato leaf curl New Delhi virus - [India-TC260-2009]	ToLCNDV-[IN-TC260-09]	KP178729	

Table S1. *Cont.*

Tomato Leaf Curl New Delhi virus isolate	Acronym	DNA-A GenBank Accession Number (DNA-A)	Strain
Tomato leaf curl New Delhi virus - [India-Faizabad-FAI-19-2010]	ToLCNDV-[IN-Fai-FAI-19-10]	KC874505	
Tomato leaf curl New Delhi virus - [India-Gwalior-GWA-5-2010]	ToLCNDV-[IN-Gwa-GWA-5-10]	KC874504	
Tomato leaf curl New Delhi virus - [India-Shahjadpur-H-SJ-2-2010]	ToLCNDV-[IN-Sha-H-SJ-2-10]	KC874502	
Tomato leaf curl New Delhi virus - [India-Jalandhar-JAL-10-2010]	ToLCNDV-[IN-Jal-JAL-10-10]	KC874509	
Tomato leaf curl New Delhi virus - [India-Modipuram-MOD-21-2010]	ToLCNDV-[IN-Mod-MOD-21-10]	KC874508	
Tomato leaf curl New Delhi virus - [India-Hisar-HIS-4-2010]	ToLCNDV-[IN-His-HIS-4-10]	KC874503	
Tomato leaf curl New Delhi virus - [India-Ash gourd-2011]	ToLCNDV-[IN-Ag-11]	JN208136	
Tomato leaf curl New Delhi virus - [India-Varanasi-2B-2011]	ToLCNDV-[IN-Var-2B-11]	KF537780	
Tomato leaf curl New Delhi virus - [India-Modipuram-MOD2-4-2011]	ToLCNDV-[IN-Mod-MOD2-4-11]	KC874507	
Tomato leaf curl New Delhi virus - [India-Deesa-DEE-1-2011]	ToLCNDV-[IN-Dee-DEE-1-11]	KC874506	
Tomato leaf curl New Delhi virus - [India-Kolkata-TC349-2011]	ToLCNDV-[IN-Kol-TC349-11]	KF551589	
Tomato leaf curl New Delhi virus - [India-Kolkata-TC350-2011]	ToLCNDV-[IN-Kol-TC350-11]	KF551590	
Tomato leaf curl New Delhi virus - [India-Junagad-TC307-2011]	ToLCNDV-[IN-Jun-TC307-11]	KF551577	
Tomato leaf curl New Delhi virus - [India-Junagad-TC309-2011]	ToLCNDV-[IN-Jun-TC309-11]	KF551576	*5
Tomato leaf curl New Delhi virus - [India-TC305-2011]	ToLCNDV-[IN-TC305-11]	KP195262	
Tomato leaf curl New Delhi virus 2 - [India-IANDS1-2011]	ToLCNDV2-[IN-IANDS1-11]	JQ897969	Other specie
Tomato leaf curl New Delhi virus 4 - [India-Junagad-TC306-2011]	ToLCNDV4-[IN-Jun-TC306-11]	KF551592	Other specie
Tomato leaf curl New Delhi virus - [India-Lucknow-Papaver somniferum-2012]	ToLCNDV-[IN-Luc-Psom-12]	KC513822	
Tomato leaf curl New Delhi virus - [India-Delhi-Cucumis sativus-2012]	ToLCNDV-[IN-Del-Csat-12]	KC545812	
Tomato leaf curl New Delhi virus - [India-Raebareli-2012]	ToLCNDV-[IN-Rae-12]	JX232220	
Tomato leaf curl New Delhi virus - [India-New Delhi-2012]	ToLCNDV-[IN-ND-12]	JX460805	
Tomato leaf curl New Delhi virus - [India-Junagadh-2012]	ToLCNDV-[IN-Junagadh-12]	KF515617	
Tomato leaf curl New Delhi virus - [India-Bhavnagar-2012]	ToLCNDV-[IN-Bhavnagar-12]	KF515616	
Tomato leaf curl New Delhi virus 3 - [India-Bijnor-Chilli-2012]	ToLCNDV3-[IN-Bij-Chi-12]	KC465466	*1
Tomato leaf curl New Delhi virus - [India-RG5-2013]	ToLCNDV-[IN-RG5-13]	KT426907	*4

Table S1. *Cont.*

Tomato Leaf Curl New Delhi virus isolate	Acronym	DNA-A GenBank Accession Number (DNA-A)	Strain
Tomato leaf curl New Delhi virus - [India-RG4-2013]	ToLCNDV-[IN-RG4-13]	KT426906	*4
Tomato leaf curl New Delhi virus - [India-RG3-2013]	ToLCNDV-[IN-RG3-13]	KT426905	*4
Tomato leaf curl New Delhi virus - [India-RG2-2013]	ToLCNDV-[IN-RG2-13]	KT426904	*4
Tomato leaf curl New Delhi virus - [India-RG1-2013]	ToLCNDV-[IN-RG1-13]	KT426903	*4
Tomato leaf curl New Delhi virus - [India-pp78-2013]	ToLCNDV-[IN-pp78-13]	KJ000564	
Tomato leaf curl New Delhi virus - [India-BG1-2014]	ToLCNDV-[IN-BG1-14]	KP868764	
Tomato leaf curl New Delhi virus - [India-TN TDK CHOU2-2014]	ToLCNDV-[IN-TN TDK CHOU2-14]	KP191047	*3
Tomato leaf curl New Delhi virus - [India-New Delhi-AVT1]	ToLCNDV-[IN-ND-AVT1]	AY428769	
Tomato leaf curl New Delhi virus - [India-Lucknow]	ToLCNDV-[IN-Luc]	Y16421	
Tomato leaf curl New Delhi virus - [India-Udayapur-okra-OY142]	ToLCNDV-[IN-Uda-Okr-OY142]	EF035482	
Tomato leaf curl New Delhi virus - [Pakistan-Solanum nigrum-1997]	ToLCNDV-[PK-Sn-97]	AJ620187	
Tomato leaf curl New Delhi virus - [Pakistan-Islamabad-T1/8-2000]	ToLCNDV-[PK-Isl-T1/8-00]	AF448059	
Tomato leaf curl New Delhi virus - [Pakistan-Dargai-T5/6-2001]	ToLCNDV-[PK-Dar-T5/6-01]	AF448058	
Tomato leaf curl New Delhi virus - [Pakistan-Solanum nigrum-2004]	ToLCNDV-[PK-Sn-04]	DQ116885	
Tomato leaf curl New Delhi virus - [Pakistan-Solanum nigrum-PT10-2004]	ToLCNDV-[PK-Sn-PT10-04]	DQ116883	
Tomato leaf curl New Delhi virus - [Pakistan-Khalawal-Chili-2004]	ToLCNDV-[PK-Kha-Chi-04]	DQ116880	
Tomato leaf curl New Delhi virus - [Pakistan-Multan-Luffa-2004]	ToLCNDV-[PK-Mul-Luf-04]	AM292302	
Tomato leaf curl New Delhi virus - [Pakistan-Lahore-2004]	ToLCNDV-[PK-Lah-04]	AM258977	
Tomato leaf curl New Delhi virus - [Pakistan-Lahore-Solanum nigrum-2004]	ToLCNDV-[PK-Lah-Sn-04]	AM849548	
Tomato leaf curl New Delhi virus - [Pakistan-Lahore-2004]	ToLCNDV-[PK-Lah-04]	AM491590	*2
Tomato leaf curl New Delhi virus - [Pakistan-2006] ToLCNDV-[PK-06]	ToLCNDV-[PK-06]	EF620534	
Tomato leaf curl New Delhi virus - [Pakistan-Multan-2006]	ToLCNDV-[PK-Mul-06]	HQ658479	
Tomato leaf curl New Delhi virus - [Pakistan-Multan-Momordica-2007]	ToLCNDV-[PK-Mul-Mom-07]	AM747291	
Tomato leaf curl New Delhi virus - [Pakistan-2008]	ToLCNDV-[PK-08]	AM947506	

Table S1. *Cont.*

Tomato Leaf Curl New Delhi virus isolate	Acronym	DNA-A GenBank Accession Number (DNA-A)	Strain
Tomato leaf curl New Delhi virus - [Pakistan-Solanum-MS4-2009]	ToLCNDV-[PK-Sol-MS4-09]	FN435310	
Tomato leaf curl New Delhi virus - [Pakistan-Solanum-MS1-2009]	ToLCNDV-[PK-Sol-MS1-09]	FN435309	
Tomato leaf curl New Delhi virus - [Pakistan-Chenopodium album-Mn-05-2012]	ToLCNDV-[PK-Cal-Mn-05-12]	KC914896	
Tomato leaf curl New Delhi virus - [Pakistan-47SB-2012]	ToLCNDV-[PK-47SB-12]	KT948072	
Tomato leaf curl New Delhi virus - [Pakistan-Lahore-Convolvulus arvensis-2013]	ToLCNDV-[PK-Lah-Car-13]	KC960492	
Tomato leaf curl New Delhi virus - [Pakistan-Lahore-Rumex dentatus-2013]	ToLCNDV-[PK-Lah-Rd-13]	HG316125	
Tomato leaf curl New Delhi virus - [Pakistan-Parthenium hysterophorus-2013]	ToLCNDV-[PK-Phy-13]	KF002409	
Tomato leaf curl New Delhi virus - [Bangladesh-Jessore- Severe-2005]	ToLCNDV-[BG-Jes-Svr -05]	AJ875157	
Tomato leaf curl New Delhi virus - [Bangladesh-Chi-01-13-Tom-2006]	ToLCNDV[BD-Chi-01-13-Tom-06]	KM383744	
Tomato leaf curl New Delhi virus - [Bangladesh-Joy-01-02-Tom-2006]	ToLCNDV-[BD-Joy-01-02-Tom-06]	KM383740	
Tomato leaf curl New Delhi virus - [Bangladesh-Joy-02-03-Tom-2006]	ToLCNDV-[BD-Joy-02-03-Tom-06]	KM383738	
Tomato leaf curl New Delhi virus - [Bangladesh-Syl-01-19-Tom-2006]	ToLCNDV-[BD-Syl-01-19-Tom-06]	KM383737	
Tomato leaf curl New Delhi virus - [Bangladesh-Cucumber-2006]	ToLCNDV-[BD-cuc-06]	EF450316	Other specie
Tomato leaf curl New Delhi virus - [Bangladesh-Joy-03-07-Tom-2007]	ToLCNDV-[BD-Joy-03-07-Tom-07]	KM383739	
Tomato leaf curl New Delhi virus - [Bangladesh-Tha-01-37-Tom-2009]	ToLCNDV-[BD-Tha-01-37-Tom-09]	KM383736	
Tomato leaf curl New Delhi virus - [Bangladesh-Jam-01-40-Tom-2010]	ToLCNDV-[BD-Jam-01-40-Tom-10]	KM383743	
Tomato leaf curl New Delhi virus - [Bangladesh-Jam-02-44-Tom-2010]	ToLCNDV-[BD-Jam-02-44-Tom-10]	KM383742	
Tomato leaf curl New Delhi virus - [Bangladesh-Jes-01-33-Tom-2010]	ToLCNDV-[BD-Jes-01-33-Tom-10]	KM383741	
Tomato leaf curl New Delhi virus - [Thailand-Cucurbit-2006]	ToLCNDV-[TH-Cuc-06]	AB330079	
Tomato leaf curl New Delhi virus - [Thailand-Cucurbit-2006]	ToLCNDV-[TH-Cuc-06]	AB368448	
Tomato leaf curl New Delhi virus - [Thailand-Bottle gourd-2006]	ToLCNDV-[TH-BG-06]	AB368447	
Tomato leaf curl New Delhi virus - [Thailand-Kamphaengsaen-AFSP2c-Sauropus androgynus-2010]	ToLCNDV-[TH-Kam-AFSP2c-Sa-10]	JN809814	
Tomato leaf curl New Delhi virus - [Thailand-Luffa]	ToLCNDV-[TH-Luf]	AF102276	

Table S1. *Cont.*

Tomato Leaf Curl New Delhi virus isolate	Acronym	DNA-A GenBank Accession Number (DNA-A)	Strain
Tomato leaf curl New Delhi virus - [Taiwan-Melon-2007]	ToLCNDV-[TW-Mel-07]	GU180095	
Tomato leaf curl New Delhi virus - [Indonesia-Java-Cucumber-2008]	ToLCNDV-[ID-JV-Cuc-08]	AB613825	
Tomato leaf curl New Delhi virus - [Iran-X3A-2013]	ToLCNDV-[IR-X3A-13]	KJ778694	
Tomato leaf curl New Delhi virus - [Iran-X1A-2013]	ToLCNDV-[IR-X1A-13]	KJ778692	
Tomato leaf curl New Delhi virus - [Iran-UZ-M9-2014]	ToLCNDV-[IR-UZ-M9-14]	KP641673	
Tomato leaf curl New Delhi virus - [Iran-UZ-C6-2014]	ToLCNDV-[IR-UZ-C6-14]	KP641675	
Tomato leaf curl New Delhi virus - [Iran-UZ-T4-2014]	ToLCNDV-[IR-UZ-T4-14]	KP641677	
Tomato leaf curl New Delhi virus - [Spain-MU-8.1-Squash-2012]	ToLCNDV-[ES-MU-8.1-Sq-12]	KF749224	ES
Tomato leaf curl New Delhi virus - [Spain-MU-11.1-Squash-2012]	ToLCNDV-[ES-MU-11.1-Sq-12]	KF749225	ES
Tomato leaf curl New Delhi virus - [Spain-Alm-661-Squash-2013]	ToLCNDV-[ES-Alm-661-Sq-13]	KF749223	ES
Tomato leaf curl New Delhi virus - [Spain-Almeria-Zucchini-2013]	ToLCNDV-[ES-Alm-Zucchini-13]	KF891468	ES
Tomato leaf curl New Delhi virus - [Spain-Almeria-TomatoA4-2014]	ToLCNDV-[ES-Alm-TomA4-14]	KM977733	ES
Tomato leaf curl New Delhi virus - [Spain-Almeria-TomatoA5-2014]	ToLCNDV-[ES-Alm-TomA5-14]	KT175406	ES

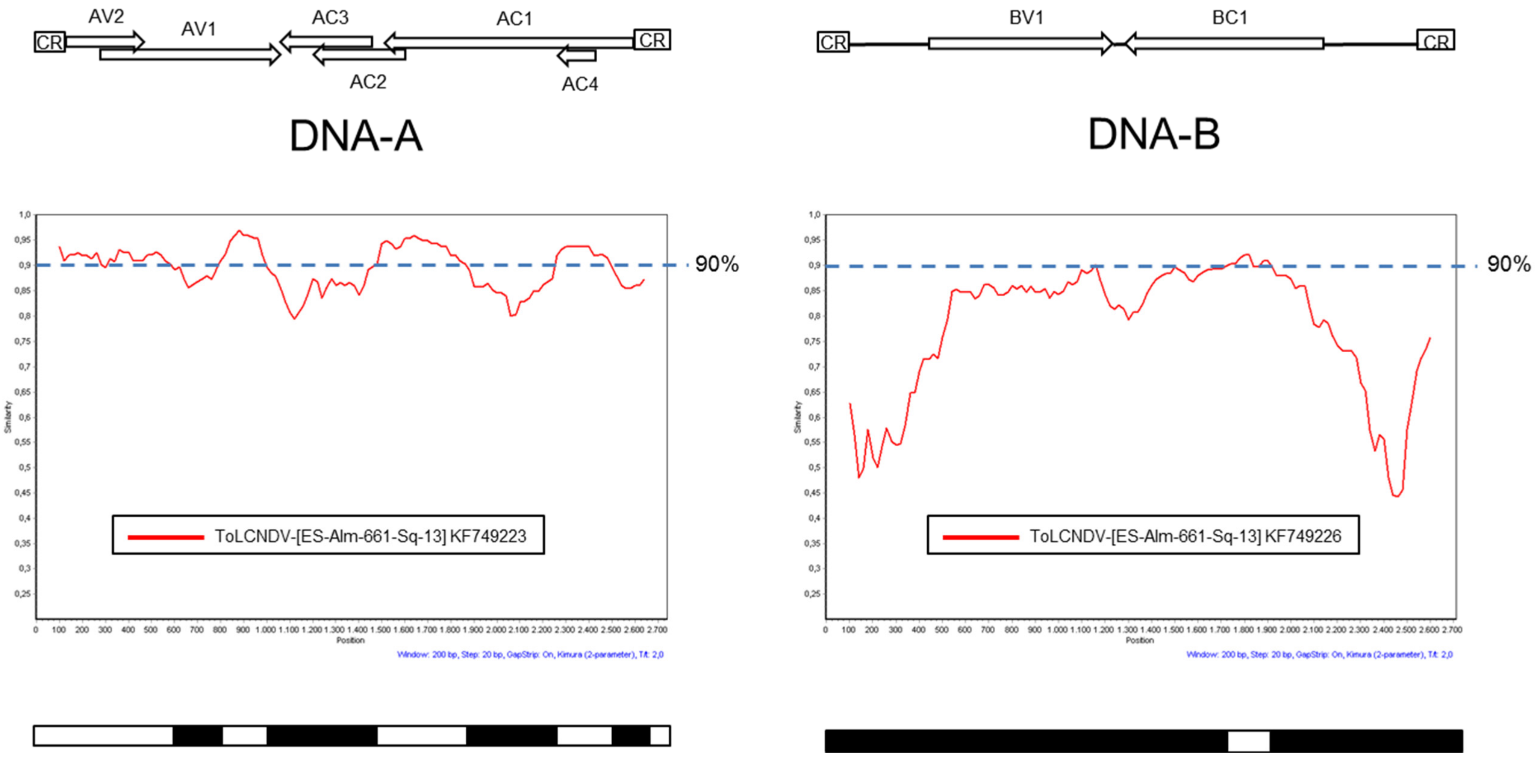

Figure S2. Plotsimilarity diagrams comparing DNA-A and DNA-B nucleotide sequences of tomato leaf curl New Delhi virus isolates. Plotsimilarity diagrams (scanning window 200 nt) comparing the nucleotide sequences of DNA-A and DNA-B of isolate [ES-Alm-661-Sq-13] of tomato leaf curl New Delhi virus (ToLCNDV-[ES-Alm-661-Sq-13]) (this study) with the corresponding ones of the ToLCNDV isolate [IN-ND-Svr-92] (ToLCNDV-[IN-ND-Svr-92]) from India (Gen Bank accession numbers U15015 and U15017 for DNA-A and DNA-B, respectively). The regions for which less than 90% identity was observed between ToLCNDV-[ES-Alm-661-Sq-13] and ToLCNDV-[IN-ND-Svr-92] are schematically shown as black boxes at the bottom of the figure. Positions of the open reading frames and the common region (CR) are indicated at the top of the figure. The horizontal broken line indicates the position for the 90% identity between the sequences compared.

© 2016 by the authors; licensee MDPI, Basel, Switzerland. This article is an open access article distributed under the terms and conditions of the Creative Commons by Attribution (CC-BY) license (http://creativecommons.org/licenses/by/4.0/).
